# Impact of Early Reoperation following Living-Donor Liver Transplantation on Graft Survival

**DOI:** 10.1371/journal.pone.0109731

**Published:** 2014-11-14

**Authors:** Yoshikuni Kawaguchi, Yasuhiko Sugawara, Nobuhisa Akamatsu, Junichi Kaneko, Tsuyoshi Hamada, Tomohiro Tanaka, Takeaki Ishizawa, Sumihito Tamura, Taku Aoki, Yoshihiro Sakamoto, Kiyoshi Hasegawa, Norihiro Kokudo

**Affiliations:** 1 Artificial Organ and Transplantation Surgery Division, Department of Surgery, Graduate School of Medicine, University of Tokyo, Tokyo, Japan; 2 Organ Transplantation Service, University of Tokyo, Tokyo, Japan; 3 Department of Gastroenterology, Graduate School of Medicine, University of Tokyo, Tokyo, Japan; University of Louisville, United States of America

## Abstract

**Background:**

The reoperation rate remains high after liver transplantation and the impact of reoperation on graft and recipient outcome is unclear. The aim of our study is to *evaluate the impact of early reoperation following living-donor liver transplantation* (LDLT) on graft and recipient survival.

**Methods:**

Recipients that underwent LDLT (n = 111) at the University of Tokyo Hospital between January 2007 and December 2012 were divided into two groups, a reoperation group (n = 27) and a non-reoperation group (n = 84), and case-control study was conducted.

**Results:**

Early reoperation was performed in 27 recipients (24.3%). Mean time [standard deviation] from LDLT to reoperation was 10 [9.4] days. Female sex, Child-Pugh class C, Non-HCV etiology, fulminant hepatitis, and the amount of intraoperative fresh frozen plasma administered were identified as possibly predictive variables, among which females and the amount of FFP were identified as independent risk factors for early reoperation by multivariable analysis. The 3-, and 6- month graft survival rates were 88.9% (95%confidential intervals [CI], 70.7–96.4), and 85.2% (95%CI, 66.5–94.3), respectively, in the reoperation group (n = 27), and 95.2% (95%CI, 88.0–98.2), and 92.9% (95%CI, 85.0–96.8), respectively, in the non-reoperation group (n = 84) (the log-rank test, p = 0.31). The 12- and 36- month overall survival rates were 96.3% (95%CI, 77.9–99.5), and 88.3% (95%CI, 69.3–96.2), respectively, in the reoperation group, and 89.3% (95%CI, 80.7–94.3) and 88.0% (95%CI, 79.2–93.4), respectively, in the non-reoperation group (the log-rank test, p = 0.59).

**Conclusions:**

Observed graft survival for the recipients who underwent reoperation was lower compared to those who did not undergo reoperation, though the result was not significantly different. Recipient overall survival with reoperation was comparable to that without reoperation. The present findings enhance the importance of vigilant surveillance for postoperative complication and surgical rescue at an early postoperative stage in the LDLT setting.

## Introduction

Continuous advances in surgical techniques, postoperative management, and immunosuppression have improved the safety of liver transplantation (LT) and patient survival [1]–[5]. In fact, overall survival rates in the later period of experience are reportedly better than those in the earlier period [6]. The Japanese Liver Transplantation Society reported overall survival rates for deceased-donor LT (DDLT) (n = 98) of 80.5% at 1 year, 77.8% at 3 years, and 76.0% at 5 years, and overall survival rates for living-donor LT (LDLT) (n = 6097) of 83.4% at 1 year, 79.3% at 3 years, and 76.9% at 5 years based on the data from 1998 to 2010 [7]. Similar survival rates are reported in Europe (83% at 1 year, and 71% at 5 years [1995–2000]) [8] and the United States (83.3% at 1 year [2002–2004], and 67.4% at 5 years [1997–2000]) [9].

Despite improved graft and recipient survival, the reoperation rate among LT recipients remains high, ranging from 9.2% to 34% [10]–[13], compared to that for liver resection, which ranges from 2.5% to 10.9% [14]–[16]. While several studies about the post-LT complication rates, including early reoperation, have been reported, there are few reports of the factors associated with early reoperation and the influence of early reoperation on LT recipient outcome. Recent reports indicate that early reoperation is a risk factor for impaired recipient outcome in both LDLT [12] and DDLT [10] recipients.

In the present study, we conducted a retrospective analysis investigating the incidence and cause of early reoperation, the factors associated with early reoperation, and the impact of early reoperation on graft and recipient survival among 111 consecutive adult LDLT recipients.

## Patients and Methods

### Patients

Between January 1996 and December 2012, 500 patients, including 77 pediatric patients, underwent LDLT at the University of Tokyo Hospital. Considering the technical standardization and establishment of criteria for reoperation, 111 consecutive adult LDLT cases between January 2007 and December 2012 were the subjects of the present study. The clinical records of these patients were retrospectively reviewed. The data of blood tests were based on the results in recipients' admission for the transplantation. All operations and reoperations were performed after obtaining informed consent from the patients and approval by the local ethics committee of the University of Tokyo.

### Graft selection criteria and surgical treatment

The indication for LDLT and the type of liver graft were determined according to the ratio of the remnant liver volume to the total liver volume in living donors, and that of the graft volume to the standard liver volume (SLV) [17] in recipients [4]. Briefly, 40% of the recipient SLV was the minimum requirement for the graft and the donor remnant liver volume needed to be over 30% of total liver volume of the donor. Our detailed donor selection criteria and surgical procedures for both the donor and recipient are described elsewhere [18].

### Postoperative management

All recipients were transferred to the intensive care unit with respirator support after the initial LDLT procedure. Recipients with an uneventful course were transferred to the surgical ward around postoperative day (POD) 5.

Routine postoperative investigations were as follows; blood tests (complete blood count, biochemical measurements, and coagulation profiles) were performed three or four times daily until POD 3, and twice daily between POD 4 and POD 14, chest and abdominal radiographs were examined twice daily until POD 3 and once daily between POD 4 and POD 14, Doppler ultrasonography to examine flow in the graft vessels was performed at least twice daily until POD 14 to detect abnormal flow in the hepatic artery/portal vein/hepatic vein, thrombi in the graft vessels, and intraabdominal fluid collection.

Indications for blood transfusions were as follows: red blood cell concentrate if the hemoglobin level or hematocrit was less than 6 g/dL and 15%, respectively, FFP if the prothrombin time-international normalized ratio (PT-INR) was greater than 2.00, and platelet concentrate if the platelet count was less than 3.0×10^4^/µL.

The basic immunosuppression regimen consisted of tacrolimus and steroids for all recipients, and the doses of each drug were gradually tapered for 6 months after LDLT. Our detailed protocol of immunosuppression is described elsewhere [2].

### Anticoagulation regimen after LDLT

To prevent early vascular thrombosis, anticoagulation therapy was started just after transplantation and continued until POD 14 in all recipients. The regimen was started with dalteparin (25 IU/kg/d), which was administered until POD 2. On POD 3, the anticoagulant drug was changed to heparin (unfractionated heparin sodium, 5000 U/d), the dose of which was adjusted to achieve a targeted activated clotting time of between 130 and 160 seconds.

### Definitions for early reoperation and early graft loss

Early reoperation in our study was defined as surgical intervention after LDLT between just after transplant and the day of initial discharge. Early graft loss was defined as graft loss occurring within 6 months after LDLT.

### Indications for early reoperation after LDLT

Indications for early reoperation were generally divided into three categories, postoperative bleeding, vessel flow problems, and biliary complications. Reoperation for postoperative bleeding was indicated for recipients with postoperative bleeding with hemodynamic instability, hemorrhage above Grade B defined by International Study Group of Liver Surgery [19], or suspected intraabdominal hematoma infection. Vessel flow problems included hepatic arterial thrombosis, portal venous thrombosis, and decreased or hepatofugal portal flow developing during the early postoperative period, all which were detected by Doppler US. Biliary complications, which we initially attempted to treat with interventional strategies, were indicated for early reoperation in cases with massive biliary leakage resulting in biliary peritonitis or biliary obstruction with intrahepatic biliary dilatation just after LDLT.

### Ethics Statement

All LDLTs were performed after individually obtaining informed consent from recipients and donors. LDLT program at the University of Tokyo Hospital has been approved by its Institutional Review Board, and all aspects of the procedures have been conducted according to the principles expressed in the Declaration of Helsinki. The current human subject research was approved as project number G3515 by Graduate School of Medicine and Faculty of Medicine, the University of Tokyo Research Ethics Committee and Human Genome, Gene Analysis Research Ethics Committee. All subjects have been properly instructed and participated by signing the appropriate informed consent paperwork. In the preparation of this manuscript, all efforts have been made to protect patient privacy and anonymity.

### Statistical analysis

Continuous variables are expressed as mean values (with standard deviations). Categorical variables are expressed as number (%), and were compared between groups using Fisher's exact test or the chi-square test, as appropriate. Graft and overall survival were measured from the time of LT. Survival curves were constructed using the Kaplan-Meier method, and compared using the log-rank test. Factors with p<0.10 in a Cox proportional hazard model as a univariable analysis were considered potential risk factors and were further analyzed in a multivariable Cox model. Hazard ratios (HR) and 95% confidential intervals (CI) were calculated for each factor. A p value of less than 0.05 was considered to indicate statistical significance. Statistical analysis was performed with JMP software (version 9.0.2; SAS Institute Inc., Cary, NC).

## Results

### Patient characteristics

The characteristics of the 111 consecutive patients are summarized in Table 1. The cohort included 50 males and 61 females (male:female, reoperation group; 7: 20, non-reoperation group; 43: 41). Mean [standard deviation (SD)] age was 51 [12] years (reoperation group; 49.9 [13.2] years, non-reoperation group; 50.7 [11.6] years). Mean [SD] Child-Pugh score was 9.3 [1.9] (reoperation group; 9.6 [1.9], non-reoperation group 9.1 [1.9]) and the mean [SD] model for end-stage liver disease score was 16.8 [7.3] (reoperation group; 17.6 [7.0], non-reoperation group; 16.6 [7.4]). There was no significant difference in the indications between the reoperation group and the non-reoperation group (Table 2). The indications for LT were liver cirrhosis caused by hepatitis C virus infection (reoperation group vs. non-reoperation group, 5 [18.5%] vs. 32 [38.1%], p = 0.052), liver cirrhosis caused by hepatitis B virus infection (2 [7.4%] vs. 13 [15.5%], p = 0.35), primary biliary cirrhosis (5 [18.5%] vs. 15 [17.8%], p>0.99), primary sclerosing cholangitis (5 [18.5%] vs. 15 [17.8%], p>0.99), alcoholic cirrhosis (2 [7.4%] vs. 5 [6.0%], p = 0.68), biliary atresia (1 [3.7%] vs. 2 [2.4%], p = 0.57), autoimmune hepatitis (1 [3.7%] vs. 2 [2.4%], p = 0.57), fulminant hepatitis (6 [22.3%] vs. 7 [8.3%], p = 0.07), and others (5 [18.5%] vs. 2 [2.4%]).

**Table 1:** Characteristics of reoperation and non-reoperation cases after LDLT.

Variables			Total (n = 111)	Reoperation Group (n = 27, 24.3%)	Non-reoperation Group (n = 84, 75.7%)
Recipient factors					
	Age, y*		50.5 [12.0]	49.9 [13.2]	50.7 [11.6]
	Sex (female), n (%)		61 (55.0)	20 (74.1)	41 (48.8)
	Child-Pugh score, pts*		9.3 [1.9]	9.6 [1.9]	9.1 [1.9]
	MELD score, pts*		16.8 [7.3]	17.6 [7.0]	16.6 [7.4]
	Preoperative status (hospitalized), n (%)		5 (4.5)	1 (3.7)	4 (4.8)
	Preoperative blood data*				
		Albumin level, g/dL	2.9 [0.4]	2.8 [0.3]	2.9 [0.5]
		Serum creatinine, mg/dL	0.8 [0.5]	0.8 [0.3]	0.8 [0.6]
		Total bilirubin, mg/dL	8.9 [9.1]	8.9 [8.5]	9.0 [9.4]
		PT-INR	1.51 [0.68]	1.60 [0.46]	1.49 [0.74]
		Platelet count, ×10^4^/µL	8.8 [6.5]	8.9 [7.3]	8.7 [6.2]
Donors factors					
	Age, years*		39.6 [12.7]	38.6 [13.5]	39.6 [12.5]
	Sex (female), n (%)		60 (54.1)	15 (55.6)	45 (53.6)
	Graft type (LL: RL:PS)		40: 67: 4	12: 14: 1	28: 53: 3
	GV/SLV, %		45.4 [9.7]	45.5 [11.1]	45.4 [9.3]
	GV, g		528 [126]	504 [129]	536 [125]
Operative factors*					
	Operative time, min		788 [132]	801 [206]	783[99]
	Operative blood loss, L		5.5 [7.9]	7.7 [15.2]	4.8 [2.7]
	Transfusion				
		Red blood cell concentrate, U	9.5 [12.3]	13.6 [22.2]	8.2 [6.2]
		Fresh frozen plasma, U	21.2 [19.2]	29.8 [33.8]	18.4 [9.8]
		Platelet concentrate, U	24.6 [19.7]	25.9 [24.5]	24.2 [18.1]
	Biliary reconstruction, duct-to-duct, n (%)		99 (89.2)	25 (92.6)	74 (88.1)

Abbreviations: LDLT, living-donor liver transplantation; LL, left lobe; MELD, model for end-stage liver disease; PT-INR, international normalized ratio of prothrombin time; PS, posterior sector; RL, right lobe; GV, graft volume; SLV, standard liver volume.* mean [standard deviation].

**Table 2:** Comparison of primary disease between reoperation and non-reoperation cases after LDLT.

Variables	Total (n = 111)	Reoperation Group (n = 27, 24.3%)	Non-reoperation Group (n = 84, 75.7%)	p value
Liver cirrhosis-HCV	37 (33.4)	5 (18.5)	32 (38.1)	0.052
Liver cirrhosis-HBV	15 (13.5)	2 (7.4)	13 (15.5)	0.35
PBC	20 (18.1)	5 (18.5)	15 (17.8)	>0.99
PSC	6 (5.4)	0 (0)	6 (7.1)	0.33
Alcoholic cirrhosis	7 (6.3)	2 (7.4)	5 (6.0)	0.68
Biliary atresia	3 (2.7)	1 (3.7)	2 (2.4)	0.57
Autoimmune hepatitis	3 (2.7)	1 (3.7)	2 (2.4)	0.57
Fulminant hepatitis	13 (11.6)	6 (22.3)	7 (8.3)	0.07
Others	7 (6.3)	5 (18.5)	2 (2.4)	N.A.

Abbreviations: HCV, hepatitis C virus; HBV, hepatitis B virus; PBC, primary biliary cirrhosis, PBC; PSC, Primary sclerosing cholangitis; LDLT, living-donor liver transplantation; N.A., not applicable. n (%).

### Profiles of early reoperation

Early reoperations after LDLT (reoperation group) were performed in 27 recipients (24.3%) on POD 10 [9.4]. Among them, 19 cases (70.4%) were performed within 10 days after LDLT and 5 cases (18.5%) required multiple reoperations. Table 3 lists the reasons for reoperation, which comprised mainly postoperative bleeding (n = 13, 48%), vessel problems (n = 8, 30%), and biliary complications (n = 5, 19%). One recipient underwent reoperation for strangulated bowel obstruction. Early graft loss subsequent to early reoperation occurred in 4 cases. Two recipients had graft loss subsequent to reoperation for portal venous thrombosis and simultaneous hepatic artery and portal venous thrombosis, and both underwent successful retransplantation. The remaining two recipients underwent reoperation for biliary problems, one for severe biliary leakage and the other for biliary stricture with severe cholangitis, both of which finally resulted in graft loss; only one recipient was saved by retransplantation.

**Table 3:** Reasons of reoperation after LDLT.

Variables		Number of cases (n = 27)	Early graft loss, n (%)
Postoperative bleeding		13	
	Graft surface	4	0
	Diaphragm	3	0
	Hepatic artery	3	0
	Hilar plate	1	0
	Drain insertion site	1	0
	Undetected	1	0
Vessels		8	
	HAT	2	0
	PVT	3	1(33)
	Simultaneous HAT and PVT	1	1(100)
	Regurgitant portal flow	1	0
	The portal steal phenomenon in APOLT	1	0
Biliary tract		5	
	Biliary peritonitis	4	1 (25)
	Biliary stenosis	1	1 (100)
Others		1	
	Incarcerated obstruction of the jejunum	1	0

Abbreviations: LDLT, living-donor liver transplantation; HAT, hepatic artery thrombosis; PVT, portal vein thrombosis; APOLT, auxiliary partial orthotopic liver transplantation.

### Risk factors for early reoperation

The results of analyses to identify risk factors for early reoperation in a Cox proportional hazard model were shown in Table 4. Recipient female sex (hazards ratio [HR] 2.63, 95% confidential intervals [CI] 1.17–6.72, p = 0.02), Child-Pugh class C (HR 2.27, 95% CI 1.04–5.29, p = 0.04), Non-HCV etiology (HR 2.44, 95%CI 1.00–7.28, p = 0.05), fulminant hepatitis (HR 2.78, 95%CI 1.02–6.49, p = 0.05), and the amount of fresh frozen plasma (FFP) administered (HR 1.01, 95% CI 1.00–1.02, p = 0.04) were demonstrated to be potential risk factors for early reoperation with p<0.10 in the univariable Cox model. Subsequent multivariable Cox model revealed that female sex (HR 2.90, 95% CI 1.18–8.27, p = 0.02) and the amount of FFP (HR 1.02, 95%CI 1.00–1.03, p = 0.03) were independent risk factors for early reoperation.

**Table 4:** Univariable and multivariable Cox proportional hazards model analysis to identify risk factors for early reoperation.

Variables			Univariable analysis			Multivariable analysis		
			HR	95% CI	p value	HR	95% CI	p value
Recipient factors								
	Age, years		0.99	0.97–1.03	0.72			
	Sex, female		2.63	1.17–6.72	0.02	2.90	1.18–8.27	0.02
	Child-Pugh, C vs A, B		2.27	1.04–5.29	0.04	1.47	0.61–3.67	0.39
	MELD score		1.01	0.96–1.06	0.57			
	Preoperative blood data							
		Albumin level, g/dL	0.87	0.37–1.99	0.75			
		Serum creatinine, mg/dL	0.71	0.22–1.47	0.43			
		Total bilirubin, mg/dL	1.00	0.95–1.04	0.97			
		PT-INR	1.17	0.67–1.62	0.50			
		Platelet count, ×10^4^/µL	1.00	0.94–1.06	0.87			
	Primary disease							
		Non-HCV vs others	2.44	1.00–7.28	0.05	1.59	0.60–4.98	0.37
		Fulminant hepatitis vs others	2.78	1.02–6.49	0.05	2.44	0.84–6.45	0.10
Donors factors								
	Age, years		0.99	0.96–1.02	0.69			
	Sex, female		1.15	0.40–1.86	0.72			
	GV/SLV, %		1.00	0.96–1.04	0.87			
	GV, g		1.00	0.99–1.00	0.18			
Operative factors								
	Operative time, min		1.00	1.00–1.003	0.58			
	Operative blood loss, L		1.00	1.00–1.00004	0.16			
	Transfusion							
		RCC, U	1.02	1.00–1.03	0.10			
		FFP, U	1.01	1.00–1.02	0.04			
		PC, U	1.00	0.98–1.02	0.74			
	Biliary duct-to-duct		1.61	0.48–9.98	0.49	1.02	1.00–1.03	0.03

Abbreviations: LDLT, living-donor liver transplantation; MELD, model for end-stage liver disease; PT-INR, international normalized ratio of prothrombin time; GV, graft volume; SLV, standard liver volume; RCC, red blood cell concentrate; FFP, fresh frozen plasma; PC, platelet concentration.

### Graft and recipient survival in each group

**Figure 1:**
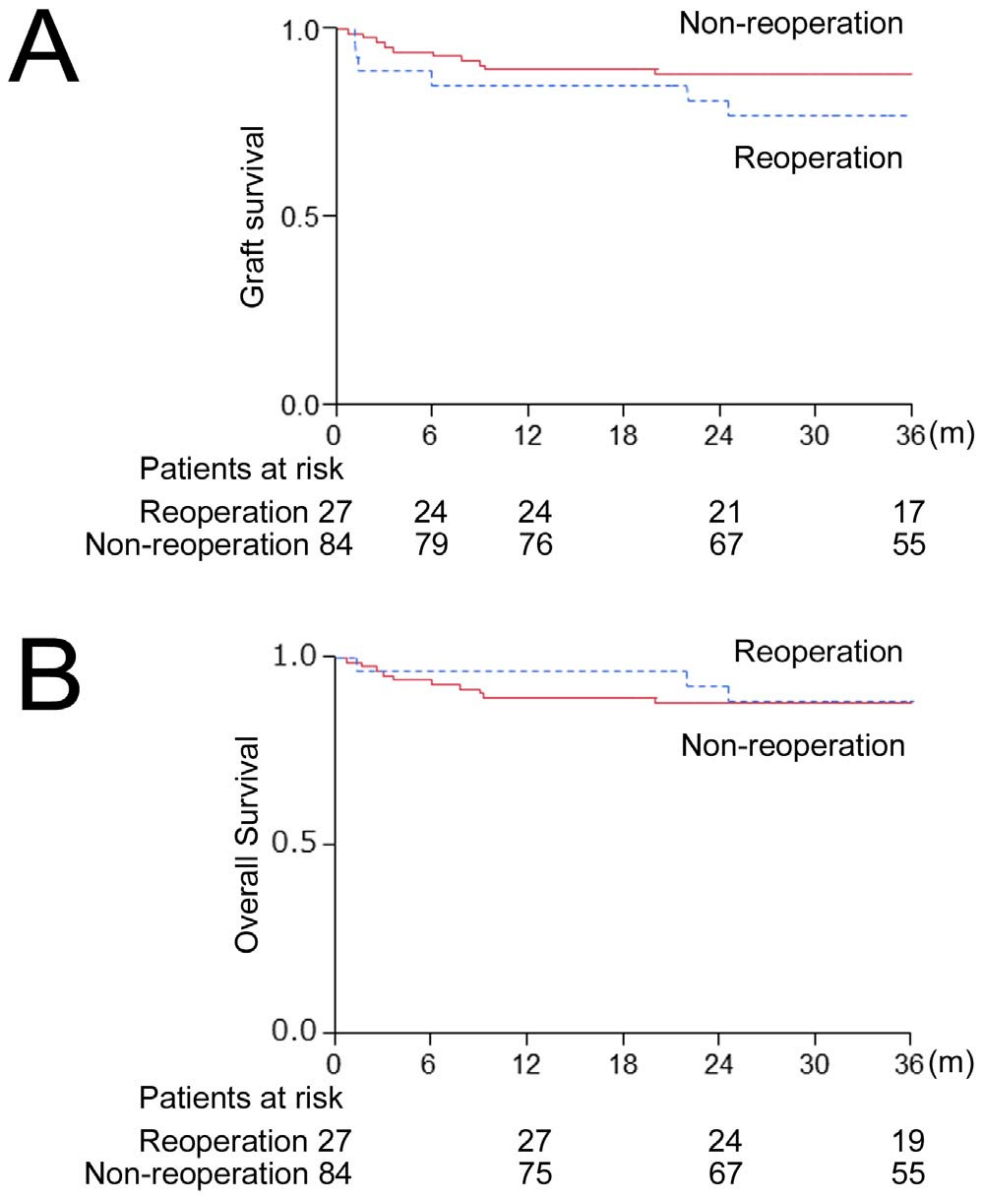
Graft and overall survival. (**A**) Graft survival rates for the reoperation group and the non-reoperation group. p = 0.31(the log-rank test). (**B**) Overall survival rates for the reoperation group and the non-reoperation group. p = 0.59 (the log-rank test).

Among the present cohort, early graft loss occurred in 10 cases, 4 in the reoperation group and 6 in the non-reoperation group. Mean follow-up time was 48.2 [25.9] months in the reoperation group and 50.6 [24.1] months in the non-reoperation group (p = 0.679). The3-, 6-, 12-, and 36- month graft survival rates were 88.9% (95%CI, 70.7–96.4), 85.2%, (95%CI, 66.5–94.3), 85.2% (95%CI, 66.5–94.3), and 77.1% (95%CI, 57.4–89.4), respectively, in the reoperation group (n = 27), and 95.2% (95%CI, 88.0–98.2), 92.9% (95%CI, 85.0–96.8), 89.3% (95%CI, 80.7–94.3), and 88.1% (95%CI, 79.2–93.4), respectively, in the non-reoperation group (n = 84). The 1- and 3-year overall survival rates were 96.3% (95%CI, 77.9–99.5), and 88.3% (95%CI, 69.3–96.2), respectively, in the reoperation group, and 89.3% (95%CI, 80.7–94.3) and 88.0% (95%CI, 79.2–93.4), respectively, in the non-reoperation group. Graft and recipient survival did not differ significantly between groups (the log-rank test, p = 0.31, and 0.59, respectively) (Figure 1). A multivariable Cox proportional hazards model was applied to evaluate the risk of early reoperation for graft survival adjusting for other potential risk factors (female sex; HR 2.67, 95%CI 1.02–8.27, p = 0.05, GV/SLV; HR 1.05, 95%CI 1.00–1.09, p = 0.06) (Table 5). Reoperation was not a significant risk factor for graft survival (HR 1.28, 95% CI 0.45–3.29, p = 0.63). No significant risk factors for overall survival were identified in a Cox proportional hazards model. The duration of postoperative hospital stay was significantly longer in the reoperation group than in non-reoperation group (99 [117] vs. 52 [29]; p<0.01).

**Table 5:** Univariable and multivariable Cox proportional hazards model analysis for graft survival.

Variables			Univariable analysis			Multivariable analysis		
			HR	95% CI	p value	HR	95% CI	p value
Reoperation			1.54	0.54–3.89	0.40	1.28	0.45–3.29	0.63
Recipient factors								
	Age, years		0.99	0.96–1.03	0.51			
	Sex, female		2.67	1.02–8.27	0.05	2.54	0.95–7.94	0.06
	Child-Pugh, C vs A, B		1.00	0.40–2.50	0.99			
	MELD score		1.05	0.99–1.11	0.14			
	Preoperative blood data							
		Albumin level, g/dL	0.92	0.33–2.44	0.87			
		Serum creatinine, mg/dL	1.36	0.66–2.12	0.34			
		Total bilirubin, mg/dL	1.03	0.98–1.07	0.28			
		PT-INR	1.23	0.69–1.70	0.40			
		Platelet count, ×10^4^/µL	1.01	0.93–1.07	0.86			
	Primary disease							
		Non-HCV vs others	1.11	0.32–2.28	0.83			
		Fulminant hepatitis vs others	2.37	0.67–6.53	0.16			
Donors factors								
	Age, years		0.95	0.38–2.38	0.90			
	Sex, female		1.15	0.40–1.86	0.72			
	GV/SLV, %		1.05	1.00–1.09	0.06	1.04	1.00–1.09	0.06
	GV, g		1.00	1.00–1.004	0.70			
Operative factors								
	Operative time, min		1.00	1.00–1.002	0.61			
	Operative blood loss, L		1.00	1.00–1.00003	0.40			
	Transfusion							
		RCC, U	0.99	0.92–1.02	0.60			
		FFP, U	1.00	0.96–1.01	0.85			
		PC, U	0.99	0.97–1.02	0.64			
	Biliary duct-to-duct		0.65	0.21–2.77	0.51			

Abbreviations: LDLT, living-donor liver transplantation; MELD, model for end-stage liver disease; PT-INR, international normalized ratio of prothrombin time; GV, graft volume; SLV, standard liver volume; RCC, red blood cell concentrate; FFP, fresh frozen plasma; PC, platelet concentration.

## Discussion

In the present study, 24% (27/111) of LDLT recipients required early reoperation, comparable to previous reports [10]–[12]. The causes of reoperation, most of which were categorized as postoperative bleeding, vascular complications, and biliary complications, were also consistent with those in previous reports [10]–[12]. Early reoperation places additional surgical stress on each recipient, which may theoretically have a negative impact on both the graft and recipient. Previous reports have indicated an impaired graft/overall survival rate of recipients with early reoperation [10], [12]. In the current study, early reoperation also tended to lead decreased graft survival rate. However, overall survival rates for recipients who underwent reoperation were comparable to those who did not, and therefore, our results enhance the importance of vigilant surveillance for early postoperative complication and early surgical rescue.

The reoperation rate after LT is reported to be high, ranging from 9.2% to 34% [10]–[13], while the reoperation rate after liver resection is reported to be as low as 2.5% to 10.9% [14]–[16]. In fact, at our institute, reoperations were performed for only 3 cases (2.7%) among 111 corresponding donors for biliary leakage (2 cases, 1.8%) and postoperative bleeding (1 case, 0.9%). The reasons for the increased rate of reoperation after LT could be attributed to poor recipient preoperative condition with hepatic failure, the administration of particular drugs such as immunosuppressants and anticoagulants, and the need for meticulous vessel reconstructions, including the hepatic vein, hepatic artery, portal vein, and bile duct [20]–[22]. The reoperation rate is reported to be even higher for LDLT than for DDLT [11], [20], [23].

Regarding risk factors for early reoperation, female sex, Child-Pugh class C, Non-HCV etiology, fulminant hepatitis, and the amount of intraoperative FFP administered were identified as possibly predictive variables, among which female sex and the amount of intraoperative FFP were identified as independent risk factors by multivariable analysis. Hendriks et al. [10] and Kappa et al. [13] reported that intraoperative blood loss predicted early reoperation. Child-Pugh class C and the amount of intraoperative FFP, which represent poor recipient liver function and have been associated with poor recipient outcome [24], [25], can reasonably be associated with early reoperation after liver transplantation, although, to the best of our knowledge, this is the first report demonstrating a higher early reoperation rate in more seriously ill recipients. Although there is no previous reports supporting the reason for female sex as a predictive risk factors of reoperation in liver transplantation, in the setting of coronary stenting, there is the preponderance of evidence supporting that female had increased risk of in-hospital death and complications [26], [27]. One possible reason in our study is that female recipient has significant smaller body and graft size in comparison with male (body height; female vs male, 156.6 [7.1] cm vs 170.5 [5.9] cm, p<0.01, body weight; 52.8 [8.4] kg vs 69.1 [9.9] kg, p<0.01, and graft size; 486.6 [127.7] g vs 579.4 [105.0] g, p<0.01), which might indicate the possible technical complications in smaller vessel reconstructions as reported in liver transplantation in children [28].

One concern for patients with liver failure and LT recipients is hemostatic balance [29]. While routine laboratory tests of these patients show bleeding diathesis, they are actually in hemostatic balance, because both pro- and antihemostatic factors are affected, the latter of which are not well reflected in routine coagulation testing [30]. This balance, however, can easily be tipped toward a hypo- or hypercoagulable state [31]. Our results demonstrating the high incidence of postoperative hemorrhage and vessel thrombosis as the cause for reoperation despite close monitoring with heparin administration, are representative of this situation. Further studies to investigate the ideal balance of coagulability are needed to reduce the incidence of early reoperation after LDLT.

Recently, Yoshiya et al. [12] of the Kyushu group and Hendriks et al. [10] reported that early reoperation was significantly associated with poor graft and/or recipient survival after LDLT and DDLT, respectively. In the present study, observed graft survival for the recipients who underwent reoperation was also lower compared to those who did not, though the result was not significantly different. Overall survival in the reoperation group was comparable to that in the non-reoperation group (Figure 1). These results in our study imply the importance of vigilant surveillance for early complications and early surgical interventions to improve graft/overall survival of recipients. However, the different results between our study (4 early graft losses [14.8%] in 27 recipients with reoperation) and the Kyushu group study (10 graft losses [34.5%] in 26 recipients with reoperation) need further investigation. The learning curve, as suggested by Kyushu group, as well as the radiologic and hematologic assays used to detect early complications, and differences in the criteria for reoperation might partially explain the discrepancy.

The main limitations of our study are its retrospective nature, the small number of cases, and biases caused by learning curves of surgical techniques and postoperative management. The early reoperation group was a small inhomogenous cohort with various causes for reoperation, which may make the data inadequate to support the findings with a multivariable analysis. Further analyses with a large number of patients in a well-designed multicenter study are needed to clarify the impact of early reoperation on outcome.

In conclusion, observed graft survival for the recipients who underwent reoperation was lower compared to those who did not undergo reoperation, though the result was not significantly different. Recipient overall survival with reoperation was comparable to that without reoperation. Independent risk factors for reoperation were recipient female sex and the amount of intraoperative FFP in our study. The present findings enhance the importance of vigilant surveillance for early postoperative complication and early surgical rescue at a postoperative period in the LDLT setting.

## Supporting Information

Checklist S1 **This study was conducted based on the STROBE statement.**
(DOC)
